# Anti-Fatigue Glasses Based on Microprisms for Preventing Eyestrain

**DOI:** 10.3390/s22051933

**Published:** 2022-03-01

**Authors:** Zichun Le, Evhen Antonov, Qiang Mao, Viacheslav Petrov, Yuhui Wang, Wei Wang, Marina Shevkolenko, Wen Dong

**Affiliations:** 1 College of Science, Zhejiang University of Technology, Hangzhou 310023, China; lzc@zjut.edu.cn (Z.L.); maoqiang@hyc.cn (Q.M.); 2112009081@zjut.edu.cn (Y.W.); 2112009025@zjut.edu.cn (W.W.); 2 Institute for Information Recording, National Academy of Sciences of Ukraine, 03113 Kiev, Ukraine; antonov@ipri.kiev.ua (E.A.); petrov@ipri.kiev.ua (V.P.); 3 Kiev City Clinical Ophthalmological Hospital “Eye Microsurgery Centre”, 03680 Kiev, Ukraine; marinashevk@ukr.net; 4 College of Environment, Zhejiang University of Technology, Hangzhou 310014, China

**Keywords:** microprism, anti-fatigue glasses, prismatic diopter, microprism fabrication, compact measuring system

## Abstract

Although microprisms have become an important medical means of strabismus treatment, related research concerning the design, fabrication, and testing of microprismatic glasses for preventing eyestrain has rarely been reported. In this study, the structure of microprismatic glasses for preventing eyestrain related to using electronic monitors, including computers and mobile phones, is introduced. A designing theory of anti-fatigue glasses with microprisms is developed. The fabrication technique and the process are described, and the performances of the fabricated microprisms are characterized. Finally, a compact testing system for the measurement of prismatic diopter is designed and constructed. This measuring system can be used not only for Fresnel microprisms, but also for other types of prisms. The measured results agree with our calculations. Although this study is focused on optimizing the objective prismatic diopter for anti-fatigue microprismatic glasses, 2.0–3.0 prismatic diopters (Δ) for each eye in the anti-fatigue glasses are suggested according to our experience on strabismus treatments. The clinical research for patients using the developed anti-fatigue glasses will be fully implemented in our further research to confirm the optimal subjective prismatic value.

## 1. Introduction

Anti-fatigue glasses can be very useful for different applications such as reading books, making precise technological operations, and working with the electronic monitors of computers and mobile phones. The last case is the most important, because many people now work with computers for two or more hours per day; this is the normal regime for working, yet is a very intensive strain on the eyes [1,2,3].

Glasses for using computers have about 60 percent of the magnifying power of general-use glasses, because computer screens are usually positioned at about 600 mm from the user’s eyes [4]. The simplest glasses for using computers have single refractive lenses, prescribed to give the most comfortable vision at the computer screen. In addition to refraction, two further eye-related problems exist: the harmful blue-violet radiation from the screen and the tension of eye muscles [5]. The most recent publications on glasses [6] and related products [7,8] for computer work mainly consider protection against harmful blue-violet light radiation emitted by the screen using different filters, which remove the blue component of the spectrum. However, studies have been rare on optical devices for protecting eyes from drying out, fatigue, or other unpleasant symptoms associated with prolonged focus on one near object, when working with electronic monitors such as computers and mobile phones [2,9].

During long periods of work with computers or mobile phones, the eye muscles constantly tense up and incline the sight lines from both eyes to a single point on the screen. It is well known that for all work connected with tension of the muscular apparatus of the eye, the probability exists of an excessive increase in intraocular pressure [10,11,12]. The inclination of the visual axes of the eyes on a close object also causes deformation of the eye, with an increase in its size in the anterior–posterior direction. Moreover, any prolonged visual stress associated with work by the eyes will lead to the deterioration of vision and in turn cause myopia. Recent studies on the psychosocial aspects of strabismus have shown that the accommodation and the convergence of the eyes are connected with an increase in pressure of the vitreous body of the eyes [13,14,15], which in turn leads to deformation of the eyeball and stretching of the meninges to promote the progression of myopia. The greater the necessary convergence of the eyes the greater the pressure increase; therefore, the greater the progression of myopia [16].

Besides the effects on the eyeball itself, to focus the beam from a close object onto the retina the two oblique muscles encircling the eyes are under the compressive force [17]. In addition, the convergence of two views from both eyes is provided by the contraction of the internal rectus muscles of the eyes. Thus, during visual work with close objects three of the six eye muscles of each eye are strained; this prolonged muscular work causes fatigue of the eyes over time.

From the above, it can be concluded that it is only possible to avoid eye fatigue during long-term work close to the eyes if the conditions can be created in which the examined near object is treated as far away. One possible method is to change the direction of the light beams from the near objects by introducing an optical element and allowing the light from the near objects to reach the eyes via parallel beams. The first efforts to reduce the stress of eye accommodation can be traced back to the late 1950s, when glasses based on the combination of a refractive lens and a prism were proposed by Utekhin [18,19]. The refractive lens is for compensating the ametropia and the prism is for preventing eyestrain. With a similar idea, an anti-fatigue solution based on a separate prism was developed by Yermoshin [20]. In this solution, the separate prism takes over the work of the eye muscles, relaxing them, and preventing spasms and probable myopia. In addition, the retina can be protected from harmful radiation by means of lens coating. However, Yermoshin’s solution has all the disadvantages of prismatic optics; high weight and low cosmetic appeal. Afterwards, Dembsky tried to use two decentering refractive lenses instead of a prism to obtain prismatic effect [21]. Recently, a company named Essilor introduced a product based on this idea. The proposed decentering lens [8] is divided into two parts; the upper part of the lens provides distance vision and the lower part of the lens has an increase of 0.6 diopters for near objects. However, unlike the prismatic glasses, this solution has difficulty in supplying sufficient diopters. Therefore, Dembsky [21] proposed a microprism instead of a traditional bulk prism to reduce the weight and size of the glasses. However, the microprismatic component in Dembsky’s glasses is attached to the lower part surface of a refractive lens by simple screws, therefore the microrelief is not protected from moisture or dust. Thus, better technical solutions are necessary to create a better design of microprismatic glasses.

**Figure 1:**
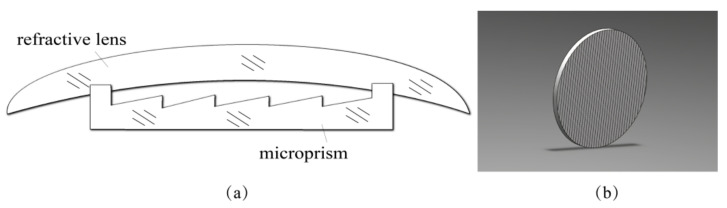
(**a**) Hermetic construction of glasses with microprisms; (**b**) diagram of a microprism.

In our previous research, we investigated the use of Fresnel prisms for strabismus treatment in children [21,22]. A totally hermetic construction of microprismatic glasses was developed to eliminate the stated disadvantages of being heavy, big, and tending to be contaminated by moisture and dust. Based on this, novel anti-fatigue glasses are proposed and the basic structure is shown in Figure 1. The microprism in the proposed anti-fatigue microprismatic glasses should be fixed to the surface of the refractive lens hermetically. To achieve this aim, a circular landing groove is made on the inner surface of the lens by a specially designed cutting tool. The microprism with a well-matched circular ledge is placed in the groove, and then the two pieces are hermetically joined by ultrasonic welding. This technology has been demonstrated in the manufacturing of traditional glasses with microprisms [23]. The refractive lens in the proposed anti-fatigue glasses can be different for the person with or without ametropia.

To the best of our knowledge, the design, fabrication, and testing of glasses with microprisms for preventing eyestrain have rarely been reported until now. In this study, the structure of microprismatic glasses for preventing eyestrain is introduced. The design theory of anti-fatigue glasses with microprisms is developed. The fabrication technique and process are described, and the performance of the fabricated microprisms is analyzed and discussed. Finally, a compact measuring system for the prismatic diopter of the prisms is designed and built. Clinical research for patients using anti-fatigue glasses is also started. It has been verified by several research participants that utilization of the proposed anti-fatigue microprismatic glasses can improve vision and relieve eyestrain.

## 2. Determination of the Parameters of Microprismatic Glasses

### 2.1. Structures of Anti-Fatigue Glasses Based on Microprisms

The function of preventing eyestrain is fulfilled by microprisms, which can change the direction of the light beams from a near object to almost parallel; therefore, the microprism is the main device in the anti-fatigue glasses. For cases without ametropia, the anti-fatigue glasses can simply be spectacles with two microprism components. However, this simple structure has the disadvantage of tending to be contaminated by moisture and dust, as stated previously. A modified structure combines a plain piece of glass and a microprism as a hermetic construction with the microrelief inside similar to the one shown in Figure 1. For cases with ametropia, a refractive lens designed to correct nearsightedness or farsightedness should be used instead of the plain glass.

For some advanced users, the anti-fatigue glasses can be designed using a more complex structure; one piece of microprism can be divided into two zones as a two-zone microprism, similar with the idea in [8,23]. The upper part is designed to meet the users’ special requirements; for example, a flat lens without any relief for resting eyes, a microprism with a special diopter for correcting a strabismus the user may have, or a microprism with a different diopter with that of the lower part for exercising the muscular apparatus of the eyes. Meanwhile, the lower part of the microprism through which a person usually looks at near objects such as for reading books or working on a computer has microrelief of the necessary prismatic diopter (2.0–3.0 Δ). This complex microprism can also be connected with a plain piece of glass or refractive lens by ultrasonic welding [23].

Although the anti-fatigue microprismatic glasses are similar to the glasses used for the therapeutic treatment of strabismus, the optimal prismatic diopter, the fabrication, and the performance characterization are still open questions.

### 2.2. Prismatic Diopter of Anti-Fatigue Glasses

To create anti-fatigue prismatic glasses the first issue is the optical center of the glasses, which is the center-to-center distance between the eyes. For cases without ametropia, the optical center is not important as the prism on the glasses is placed perpendicular to the relief strokes. However, in the presence of refractive components the features of a patient’s eyes should be considered, including the center-to-center distance. Generally, the optical center can be decided by the pupil distance data.

The most important parameter for anti-fatigue prismatic glasses is the prismatic diopter. Utekhin’s glasses were made with a total prism strength of 7.5 diopters (Δ) for both eyes [19], i.e., 3.75 Δ for each eye. The currently available Yermoshin glasses have strengths of 0.25–0.50 Δ according to their advertising information. However, it seems that these glasses are made with refractive optics and not with prisms. Dembsky’s microprismatic glasses were made of microprisms with 7.0 Δ for each eye and the value of the prismatic diopter was obtained from the consideration of the geometrical optics.

**Figure 2:**
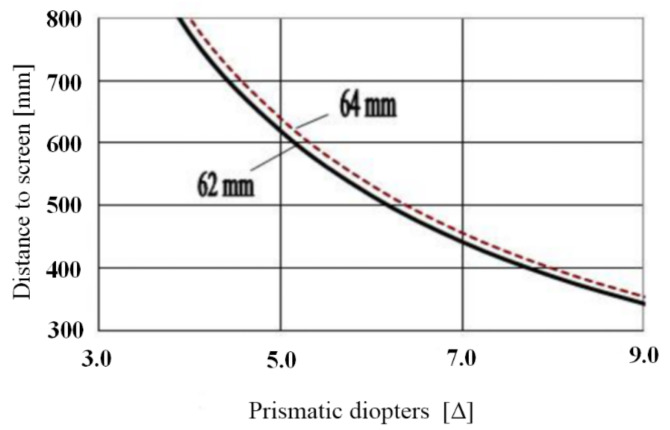
Objective prismatic diopter for anti-fatigue glasses from the consideration of geometrical optics.

The standard viewing angle of objects on a computer screen at a viewing distance of 350 mm is about 5.06–5.22° for a pupil distance of 62–64 mm, which is a typical value of pupil distance for adults. The calculated values of the necessary prismatic diopter, according to the geometrical optics and the characterization of optical materials with the refractive index of 1.46–1.52, are shown in Figure 2. For these calculated prismatic diopters, the light beams are converged exactly to the screen.

However, with the anti-fatigue microprismatic glasses the light beams are focused on the screen not only according to the prism action, but also due to the fusion provided by the human brain. Therefore, the optimal prismatic diopter should be defined as two different assessed values: the objective diopter and the subjective diopter. The objective prismatic diopter can be calculated according to geometrical optics; this is the subject of the current study. For the subjective prismatic diopter it is necessary to carry out detailed clinical studies with patients, which is beyond the scope of this paper, although we have already started the related clinical study. We think that the optimal value of the prismatic diopter in the anti-fatigue glasses can be obtained only after these precise clinical investigations. In our opinion, this prismatic diopter value should be in the range of 2.0–3.0 Δ for each eye.

### 2.3. Physical Phenomena with the Anti-Fatigue Microprismatic Glasses

**Figure 3:**
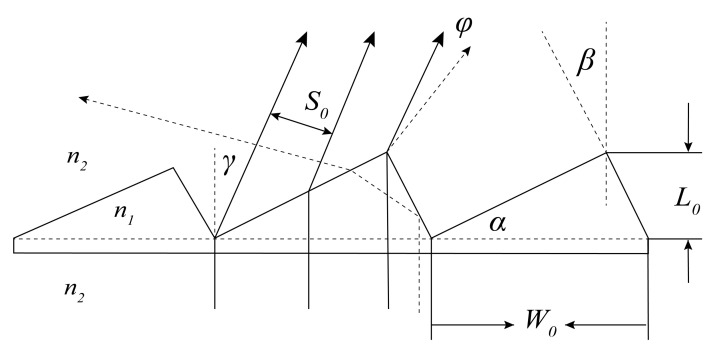
Schematic diagram of a microprism with three prism units.

The main problem with microprisms in anti-fatigue glasses is the diffraction at the microrelief that affects the visual images. The other physical disadvantage is the chromatism of white light [23]. For microprisms with a small prismatic diopter of 2.0–3.0 Δ the chromatism can be neglected, because the chromatism zone is very small and involves little beam expansion for the observation distance of 350–400 mm. A schematic diagram of a microprism with three prism units is shown in Figure 3. The parameters related to the single prism unit shown in Figure 3 are: *W*_0_ stands for the length of the bottom of the prism unit (i.e., the pitch width), *L*_0_ stands for the height of the prism unit, *α* and *β* are the angles defining the prism shape, and *n*_1_ and *n*_2_ are the refractive indexes of the microprism and the surroundings, respectively. The parameter that decides the prismatic diopter of the prism is *α*, and *β* is related to the technological requirement of reducing stray light. According to our previous experience [22], *β* should be set as 2°–3° for reducing stray light.

For the consideration of diffraction phenomena, the microprism can be treated as a blazed grating and the visual image through it will be affected by the single-slit diffraction from every single prism unit and the multiple-diffractive-beam interference from the whole microprism. The intensity of diffraction from a single prism unit can be described as [24]:

(1) $$I_{d}=I_{0}{\left(\frac{\sin a}{a}\right)}^{2}\;,\;\;a=\frac{\pi S_{0}}{\lambda }\sin\varphi ,$$

where *φ* stands for the diffractive angle, *λ* is the wavelength of incident radiation, *S*_0_ is the width of the diffractive slit when a single prism unit is considered, *I*_0_ is the initial optical intensity, and ${\left(\frac{\sin a}{a}\right)}^{2}$ is typically referred to as the single-slit diffraction factor.

The intensity of multiple-diffractive-beam interference from the whole microprism can be described as [24]:

(2) $$I_{i}=I_{0}{\left(\frac{\sin Nb}{\sin b}\right)}^{2}\;,\;b=\frac{\pi D_{1}}{\lambda }\sin\varphi ,$$

where *N* stands for the number of prism units, *D*_1_ is the width of the interference slit between two diffractive beams from prism units, and ${\left(\frac{\sin Nb}{\sin b}\right)}^{2}$ is typically referred to as the interference factor between multiple slits.

Therefore, the optical intensity through the microprism can be described as:

(3) $$I_{\mathrm{total}}=I_{0}{\left(\frac{\sin a}{a}\right)}^{2}\cdot {\left(\frac{\sin Nb}{\sin b}\right)}^{2}.$$

When *β* = 0, we get $D_{1}=W_{0}\cos\gamma$ and $S_{0}=W_{0}(\cos\;\gamma -\tan\;\alpha \;\sin\;\gamma )$, where γ is the refractive angle when paralleled light is shone onto the microprism. We then obtain:

(4) $$I_{\mathrm{total}}=I_{0}{\left(\frac{\sin\left[\frac{\pi W_{0}\left(\cos\gamma -\tan\alpha \sin\gamma \right)}{\lambda }\sin\varphi \right]}{\frac{\pi W_{0}\left(\cos\gamma -\tan\alpha \sin\gamma \right)}{\lambda }\sin\varphi }\right)}^{2}\cdot {\left(\frac{\sin\left(\frac{N\pi W_{0}\cos\gamma }{\lambda }\sin\varphi \right)}{\sin\left(\frac{\pi W_{0}\cos\gamma }{\lambda }\sin\varphi \right)}\right)}^{2}.$$

When β≠0, $D_{1}=W_{0}\cos\gamma$, and $S_{0}=W_{0}(\cos\gamma -\frac{\sin\alpha \;\cdot \;\sin\left(\gamma +\beta \right)}{\cos\left(\alpha -\beta \right)})$ we then obtain:

(5) $$I_{\mathrm{total}}=I_{0}{\left(\frac{\sin\left[\pi W_{0}\left(\cos\gamma -\frac{\sin\alpha \;\cdot \;\sin\left(\gamma +\beta \right)}{\cos\left(\alpha -\beta \right)}\right)\frac{\sin\varphi }{\lambda }\right]}{\pi W_{0}\left(\cos\gamma -\frac{\sin\alpha \;\cdot \;\sin\left(\gamma +\beta \right)}{\cos\left(\alpha -\beta \right)}\right)\frac{\sin\varphi }{\lambda }}\right)}^{2}\cdot {\left(\frac{\sin\left(\frac{N\pi W_{0}\cos\gamma }{\lambda }\sin\varphi \right)}{\sin\left(\frac{\pi W_{0}\cos\gamma }{\lambda }\sin\varphi \right)}\right)}^{2}.$$

**Figure 4:**
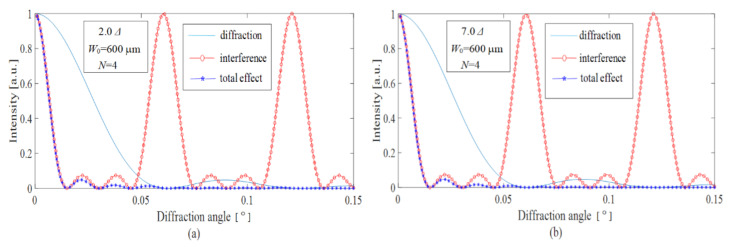
Diffraction, interference, and total effect for the microprism with *W*_0_ = 600 μm, and with 2.0 Δ for (**a**) and 7.0 Δ for (**b**).

**Figure 5:**
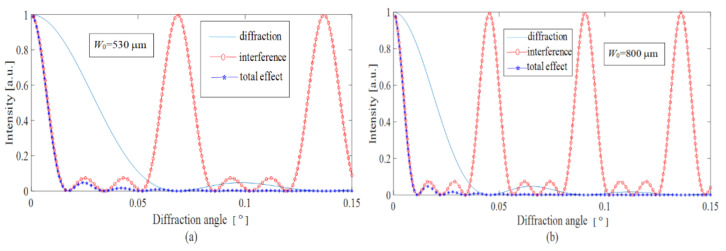
Diffraction, interference, and total effect for the microprism with 4.0 Δ and with *W*_0_ = 530 μm for (**a**) and *W*_0_ = 800 μm for (**b**).

According to the equations derived above, the simulations are performed and the obtained results are shown in Figure 4 and Figure 5. The explanations of some initial simulated parameters are as follows: Firstly, a brief analysis shows that for a lower pitch width of *W*_0_ ~ 100–200 μm the expansion of optical images after the microprism became very large (Δ*γ* ~ 0.2°–0.4°) compared with the prism deflection angle *γ* ≈ 5.2° for a pupil distance of 64 mm and an observation distance of 350 mm. The smallest resolution angle of a normal eye is usually 0.017°, therefore *W*_0_ should be around 600 μm. The value *N* is defined by the size of the microrelief area, which is illuminated by a laser beam. In our calculations, if the typical value of the pitch width is *W*_0_ = 600 µm, then *N* = 4 is a reasonable value that corresponds to the diameter of the laser beam. The working wavelength *λ* is chosen as 6328 nm.

Figure 4 shows the optical performance of the microprisms with different prismatic diopters from 2.0 Δ to 7.0 Δ and with a fixed pitch width of 600 μm. It is seen that the diffraction curves are practically unchanged for the objective prismatic diopters of 2.0–7.0 Δ. As we know, the diffraction curves are mainly determined by the effective grating period of $D_{1}=W_{0}\cos\gamma$. For lower angles of *γ*, $W_{0}\cos\gamma \approx W_{0}$; therefore, the lower values of 2.0–3.0 Δ are recommended by considering the eye image fusion carried out by the human brain.

Figure 5 shows the diffractive deflection of light for different values of pitch width and a fixed prismatic diopter of 4.0 Δ. We calculated the diffractive deflection for pitch widths in the range 300–1000 μm. The results are listed in Table 1. From Table 1 and Figure 5, we can see that the deflection of light is mainly affected by the diffraction from the single prism unit, which in turn is affected by the prismatic diopter of the prism. When *W*_0_ = 530 μm, the corresponding angle is just 0.017°; therefore, the pitch width *W*_0_ should be larger than 530 μm. However, if *W*_0_ is too large the anti-fatigue microprismatic glasses will be inconvenient for wearing due to visible streaking of the images.

Thus, the optimal microrelief pitch width for the anti-fatigue microprismatic glasses is better set as *W*_0_ = 600–800 μm. For lower pitches the microprisms would distort the observed optical images. For larger pitches the anti-fatigue glasses would not be attractive from a cosmetic viewpoint because the structures of the microrelief become clearly visible.

## 3. Fabrication of Anti-Fatigue Microprismatic Glasses

Microprism is a kind of micro-structured optical component. Usually, it can be fabricated by micro-fabricated technologies such as lithography, etching, and laser direct writing. However, for the case of microprisms the micro-fabricated techniques mentioned above are infeasible because there are slant angles related with the structure of microprisms. Therefore, a stamper with specially designed prismatic microrelief was manufactured by ultrahigh precision machining for meeting the requirements, including the accuracy of structure parameters and the optical surface of a microprism. Moreover, considering the feasibility of the mass production of microprisms, optical thermoplastic materials are the most suitable materials for actually manufacturing the microprisms by hot pressing or injection molding technology.

According to their material characteristics, polymethylmethacrylate (PMMA), polyvinylchloride (PVC), and polycarbonate (PC) are suitable materials for fabricating the anti-fatigue microprismatic elements for glasses; of these PMMA is the best, because it has a smaller chromatic dispersion, a suitable refractive index (~1.492), higher light transmissivity (90–92%), and better thermoplasticity [24]. The disadvantage of PMMA is its lower surface hardness, which therefore means the microrelief seems easier to wear. This is also a reason we use a hermetic construction for the proposed anti-fatigue microprismatic glasses.

**Figure 6:**
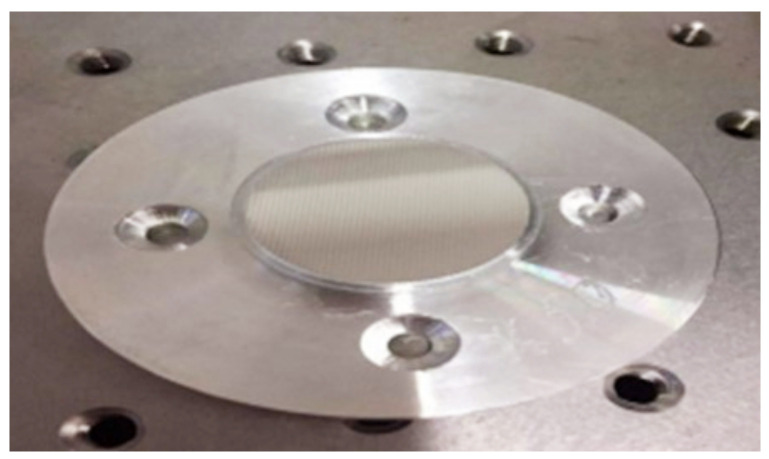
Photograph of the pressing stamper with a prismatic diopter of 5.0 Δ.

The microprisms were fabricated by hot pressing technology in this study. A pressing stamper was designed according to the results of the theoretical simulation of the microprism parameters by the method introduced above. The prismatic microrelief was then fabricated upon an extra super duralumin alloy (V-95) at the Institute for Information Recording, the National Academy of Sciences of Ukraine [21]. The fabrication of the stamper becomes more and more difficult with the smaller prismatic diopter, because angle *α* of the prism unit is increasingly smaller. Figure 6 shows a photo of the fabricated pressing stamper with a prismatic diopter of 5.0 Δ.

**Figure 7:**
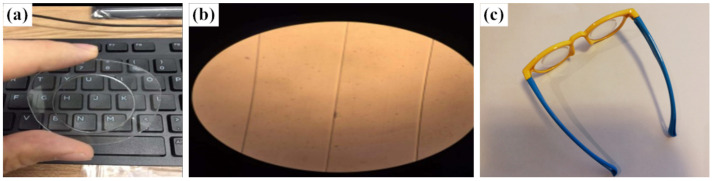
(**a**) Photograph of a sample of the PMMA microprism we fabricated; (**b**) partially enlarged view of the microrelief under an optical microscope; (**c**) photograph of the fabricated PMMA anti-fatigue glasses based on microprisms for preventing eyestrain.

The PMMA microprisms were fabricated by a servo hot pressing machine (DHF05-1T, made in China), which has a cooling system for a better molding performance. A laser-cutting machine used for obtaining a specific shape of PMMA substrate was also used. First, the pressing stamper was put on the cut and then the cleaned PMMA substrate; together they were placed in the lower mold of the servo hot pressing machine and preheated to 60–70 °C. Then, the upper mold of the servo hot pressing machine was heated to 150–165 °C and pressed on the pressing stamper with a force of 100–200 kg for 30 s. Afterwards, the cooling system was turned on in order to decrease the temperature to 60 °C under the condition of maintaining constant pressure. Finally, the cooling system was turned off, the pressure was released, and we obtained the PMMA microprismatic element. Figure 7a shows a photo of the PMMA microprism we fabricated. The general quality of the fabricated PMMA microprism was checked by an optical microscope; there was no visible damage and the structure of the microrelief was clear. Figure 7b,c shows the fabricated PMMA anti-fatigue glasses based on microprisms for preventing eyestrain.

**Figure 8:**
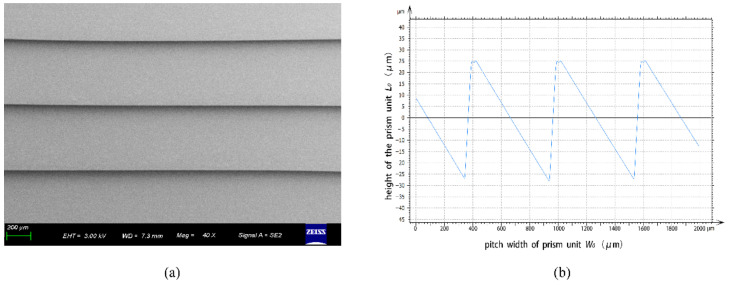
Measured results for the structure of a fabricated PMMA microprism by SEM (**a**) and by stylus profiler (**b**).

To inspect the quality and the accuracy of the fabricated microrelief on the microprism, a scanning electron microscope (SEM, ZEISS) and a stylus profiler system (Bruker, Dektak XT-E) were used to measure the structure parameters of the microrelief. Figure 8a,b show the measured results by SEM and by the stylus profiler, respectively. In addition, the measured results show that the fabricated microprism has a high-quality optical surface and very accurate structure sizes. The measured parameters by the stylus profiler include the pitch width (*W*_0_) and the height of the prism unit (*L*_0_). On the basis of the measured results we can also characterize the surface finish (*R_a_*) of the fabricated microprism by numerical analysis. The surface finish (*R_a_*) can be calculated by the contour arithmetic mean deviation:

(6) $$R_{a}=\frac{1}{m}\sum_{i=1}^{m}\left|h\right|,$$

where *m* stands for the sampling number within a sampling length, *i* is a positive integer, and *h* is the absolute value between the specific point on the contour and the reference point on the datum plane. Table 2 shows the measured results of the pitch width and the height of the prism unit, as well as the obtained surface finish results. The measured results show that the fabricated parameters basically match our design.

## 4. Measuring System of the Prismatic Diopter

The prismatic diopter is the most important parameter of the developed anti-fatigue microprismatic glasses. However, to the best of our knowledge there is no measuring system for testing the prismatic diopter of a prism or a microprism. Although we can indirectly obtain the prismatic diopter of a prism by measuring its structural parameters, it is necessary to develop a measuring system for directly testing the prismatic diopter of prisms. According to the theory introduced in Section 2, the prismatic diopter of a prism (in prismatic diopters Δ) can be easily obtained by measuring the deflection of a laser beam through it. A beam deflection (*l*_E_) of 10 mm on the screen with a distance (*L*_E_) of 1000 mm from the prism to the screen corresponds to a prismatic diopter of 1.0 prismatic diopter (Δ); therefore, for any deflection angle *γ* the prismatic diopter can be easily calculated as Δ = 100 (*l*_E_ /*L*_E_) [21].

To minimize the size of the measuring setup, it is possible to use a special scheme with a rotating frame with microprisms. We take the deflection angle (*γ*_max_) corresponding to the largest prismatic diopter as the reference value; therefore, the rotation angle should be increased depending on the increase in prism strength for prisms with smaller Δ.

**Figure 9:**
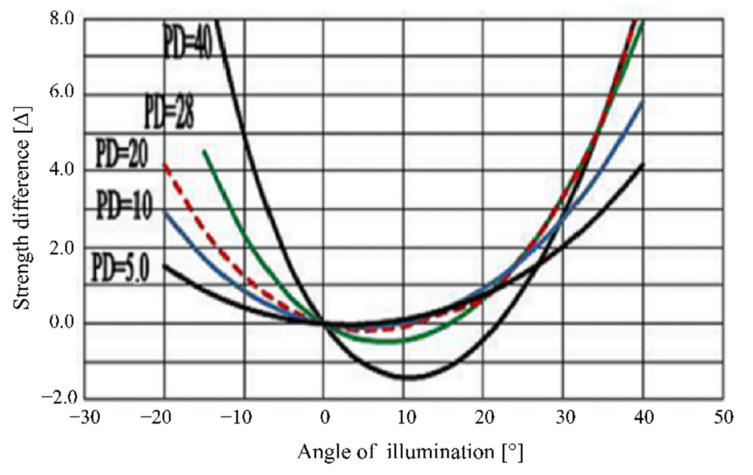
Dependence of prismatic diopter on the difference in rotating angle for prisms with different prismatic diopter (PD).

A microprism with 30.0 Δ has a deflection angle of *γ*_30_ = 16.69924° according to the definition of prismatic diopter. In our measuring system, the prism with 30.0 Δ is taken as the standard prism with the largest value of *γ*_max_; therefore, for the microprism with 30.0 Δ the rotation angle of the rotating element is set to zero (0°). For all other microprisms, the values of the rotation angle (*θ*) should be obtained for which the deflection angle *γ* = *γ*_30_ = 16.69924°, depending on the prismatic diopter for all tested microprisms. The dependence of the prismatic diopter on the rotation angle *θ* was investigated [21,22] and shown in Figure 9.

**Figure 10:**
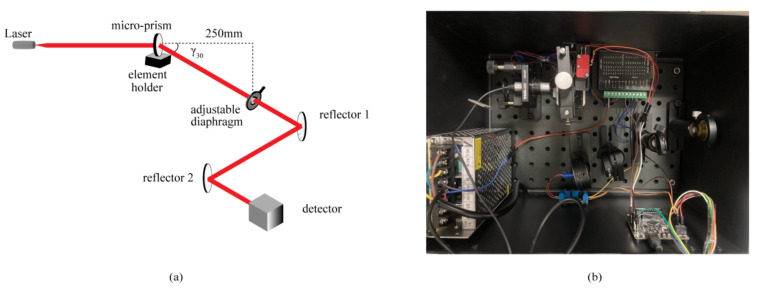
(**a**) Scheme of the developed compact measuring system for testing the prismatic diopter of prisms; (**b**) photograph of the developed compact measuring system.

A compact measuring system for obtaining the prismatic diopter of prisms was developed using the left part of the curves in Figure 9 relative to their zero position, because the curves have better linearity in this part and thus the measurement will be more accurate. The developed compact measuring system is shown in Figure 10. Figure 10a shows the scheme of the optical system; two reflectors were used in the measuring system to reduce *L*_E_ to 250 mm and in turn reduce the size of the measuring system. The adjustable diaphragm in front of the first reflector must be set in the direction of *γ*_30_, because we took the largest prismatic diopter as the reference value. In addition, the laser must exit horizontally and incident on the flat surface without microrelief to avoid measurement error; moreover, the element holder can only be rotated in one direction to improve the measuring accuracy. Figure 10b displays the photo of the developed compact measuring system for prismatic diopters of prisms or microprisms.

We measured the five fabricated samples using the developed compact measuring system; the measured results are shown in Table 3. We also analyzed the measuring error and found that it is smaller than 0.25 Δ for all the microprisms with different prismatic diopters.

We calculated and analyzed the dependence of the value of *γ* on the rotation angle *θ* for all prisms with prismatic diopters of 1.0–30.0 Δ. From the calculation results, it follows that for very low strengths of 0.5–2.0 Δ it is not possible to achieve the deflection angle of *γ*_30_ = 16.69924° by rotation of the prism, because the reflection angle of the laser beam inside the prisms exceeds the critical reflection angle in this case [24], which for PMMA with refractive index of 1.492 is calculated to 39.74° [21]. It means the beam is totally reflected inside the prism and cannot be deflected to the stated angle of *γ*_30_ = 16.69924°. For example, for a prism with a prismatic diopter of 1.0 Δ the largest possible rotation angle is *θ* = 77.76°, which corresponds to a deflection angle (*γ*) of 10.66°. For a prism with a prismatic diopter of 2.0 Δ the largest possible rotation angle *θ* = 72.598°, which corresponds to a deflection angle (*γ*) of 14.94°.

**Figure 11:**
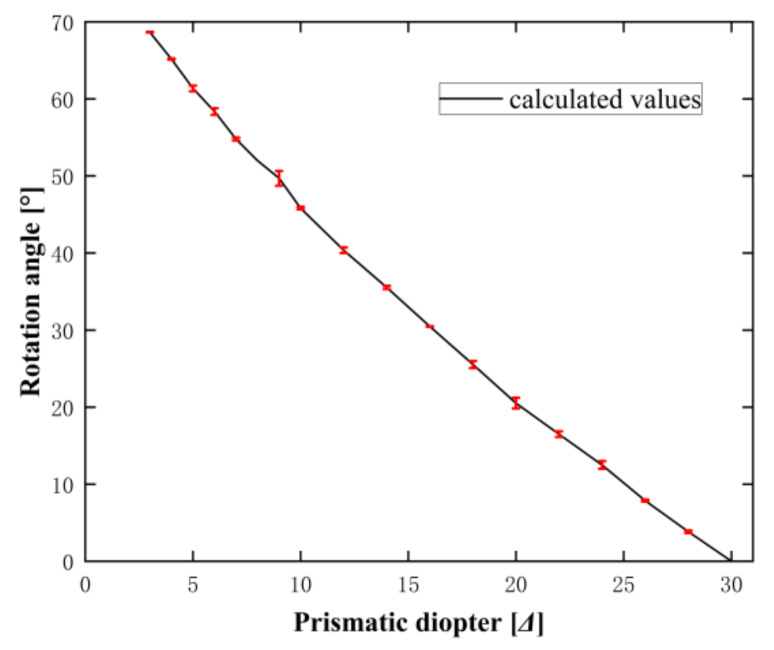
Dependence of rotation angle on the prismatic diopter of a prism.

Thus, the measuring system developed in this study can only efficiently measure the prismatic diopter of prisms within a range of 3.0–30.0 Δ. We measured microprisms with prismatic diopters from 3.0 Δ to 30.0 Δ using the developed measuring system and compared the measured results with our theoretical calculations, finding that they agreed well (see Figure 11; the measured results were shown with the error bar).

## 5. Conclusions

The possibility of using Fresnel microprismatic glasses for preventing eyestrain was thoroughly investigated. Firstly, the construction of the anti-fatigue glasses was introduced. Next, the design theory of the anti-fatigue glasses with microprisms was developed and the simulations were performed, which demonstrated the effectiveness of the proposed anti-fatigue microprismatic glasses.

Subsequently, the fabrication technique and the process were described and the general quality of the fabricated microprisms was tested by an optical microscope. In addition, the structural parameters of the microprisms were measured by a stylus profiler system and a scanning electron microscope. The measured results demonstrated the fine accuracy and the optical surface of the fabricated microprisms.

Finally, a compact testing system for measuring the prismatic diopter of the prisms was designed and built. The prismatic diopters of the fabricated microprisms were measured by the developed measuring system and the measured results agreed with the calculations. The compact measuring system we developed for the measurement of the prismatic diopter can be used for any kind of prism.

Although clinical research for patients using anti-fatigue glasses has already started, in this paper we focused on the optical theory and the techniques related to the fabrication and the measurement of the microprisms. The optimal value of the prismatic diopter of prisms in the anti-fatigue microprismatic glasses can only be obtained after precise clinical investigations. In our opinion, this value should be within the range of 2.0–3.0 prismatic diopters for each eye to help the brain achieve the total necessary convergence of the eyes for viewing near objects.

## Figures and Tables

**Table 1:** Deflection angles of light varying with different values of pitch width.

Pitch Width (*W*_0_, μm)	Angle Related to Diffraction Only (°)	Angle Related to Interference Only (°)	Angle with Total Effect (°)
300	0.118	0.03	0.032
400	0.088	0.022	0.022
500	0.071	0.018	0.019
530	0.066	0.017	0.017
600	0.061	0.015	0.015
700	0.05	0.013	0.013
800	0.045	0.011	0.012
1000	0.035	0.0087	0.0098

**Table 2:** Measured pitch width (*W*_0_), height of prism unit (*L*_0_), and surface finish (*R_a_*).

Tested Samples	Pitch Width (*W*_0_, μm)	Height of Prism Unit (*L*_0_, μm)	Surface Finish (*R_a_*, nm)
pressing stamper	600.55	54.53	147.41
microprism 1#	600.72	54.29	132.77
microprism 2#	600.62	55.84	165.15
microprism 3#	600.12	55.21	134.61
microprism 4#	600.09	55.32	126.28
microprism 5#	600.23	55.50	173.86

**Table 3:** Measured prismatic diopters of five fabricated microprisms.

Tested Samples	Rotating Angle (*θ*, °)	Prismatic Diopter (Δ)
microprism 1#	61.34	5.0
microprism 2#	61.45	5.0
microprism 3#	61.28	5.0
microprism 4#	61.17	5.0
microprism 5#	61.35	5.0

## Data Availability

Not applicable.
